# Named-Entity-Recognition-Based Automated System for Diagnosing Cybersecurity Situations in IoT Networks

**DOI:** 10.3390/s19153380

**Published:** 2019-08-01

**Authors:** Tiberiu-Marian Georgescu, Bogdan Iancu, Madalina Zurini

**Affiliations:** Department of Economic Informatics and Cybernetics, The Bucharest University of Economic Studies, 6 Piata Romana, 010374 Bucharest, Romania

**Keywords:** CVE, IoT, domain ontology, NER, cybersecurity

## Abstract

The aim of this paper was to enhance the process of diagnosing and detecting possible vulnerabilities within an Internet of Things (IoT) system by using a named entity recognition (NER)-based solution. In both research and practice, security system management experts rely on a large variety of heterogeneous security data sources, which are usually available in the form of natural language. This is challenging as the process is very time consuming and it is difficult to stay up to date with the constant findings in the areas of security threats, vulnerabilities, attacks, countermeasures, and risks. The proposed system is conceived as a semantic indexing solution of existing vulnerabilities and serves as an information tool for security management experts. By integrating the proposed system, the users can easily discover the potential vulnerabilities of their IoT devices. The proposed solution integrates ontologies and NER techniques in order to obtain a high rate of automation with the scope of reaching a self-maintained and up-to-date system in terms of vulnerabilities and common exposures knowledge. To achieve this, a total of 312 CVEs (common vulnerabilities and exposures) specific to the IoT field were identified. CVEs are arguably one of the most important cybersecurity resources nowadays, containing information about the latest discovered vulnerabilities. This set is further used as data corpus for an NER model designed to identify the main entities and relations that are relevant to IoT security. The goal is to automatically monitor cybersecurity information relevant to IoT, and filter and present it in an organized and structured framework based on users’ needs. The taxonomies specific to IoT security are implemented via a domain ontology, which is later used to process natural language. Relevant tokens are marked as entities and the relations between them identified. The text analysis solution is connected to a gateway which scans the environment and identifies the main IoT devices and communication technologies. The strength of the approach proposed within this research is that the designed semantic gateway is using context-aware searches in the modeled IoT security database and can identify possible vulnerabilities before they can be exploited.

## 1. Introduction

Although the IoT (Internet of Things) term was mentioned for the first time in 1999 [1], IoT technologies have been used for decades [2]. However, the general public has seldom had access to IoT solutions, as they were mainly used by large organizations. The development of IT technologies, especially those related to the speed of data transmission, have facilitated the introduction of many IoT devices that are now used by the general public. International standards and protocols have been established and products manufactured by different companies have been linked to each other. Taking this into account, people have begun to show an increased interest in the field of IoT in recent years, as presented in Reference [3]. Now that the Internet has become a mission-critical component of modern business, cybersecurity is considered an indispensable component of information systems [4].

IoT uses dedicated technologies designed to facilitate the development of specific solutions as well as existing technologies from other IT domains. As a rule, IoT systems are big consumers of computational resources, memory resources, and bandwidth. Therefore, existing approaches [5] for software solutions are not proven to be reliable for IoT systems also [3]. In this paper, we focus on IoT vulnerabilities found in IoT-specific technologies. To reach the proposed objective, we gathered the main data regarding IoT-specific vulnerabilities. This was further used as a training set for the named entity recognition (NER) solution described in Section 4.

The objective of the current research was inspired by the aggregation of ongoing changes in information and knowledge found within common vulnerabilities and exposures (CVEs) [6] in order to achieve a content-based extraction of the most appropriate CVEs concerning the current situation of each IoT system. A domain-based semantic annotator offers an intermediate layer between a wide selection of unstructured data found in the CVEs database and a particular implementation of an IoT system. Our approach is designed to be updated as soon as new entries in the CVEs database are made, resulting in an up-to-date framework used for extracting valuable information according to the user’s need.

The paper is structured in seven sections, starting from the basics of security vulnerabilities and leading to an example of integrating the obtained model within a semantic security gateway. Section 2 presents the literature review and emphasizes the strengths of the work within this paper compared to related works. Section 3 deals with the description of security vulnerabilities for IoT technologies, describing the CVE system that provides a reference method for publicly known vulnerabilities and exposures. From the database of all the important IT vulnerabilities reported so far, a selection of the most relevant CVEs was performed, using keyword-based searches related to IoT standards, technologies, and products. A sub-set consisting of 312 CVEs was conducted, grouped by 33 key terms. Section 4 reveals an NER-based approach for the automatic gathering of cybersecurity data of IoT devices using a semantic indexing solution. The solution uses entity recognition instruments for text analysis because most of the information about known vulnerabilities is provided as text that is only suitable for security experts and cannot be easily understood or directly used by automated security systems [7]. The architecture of the solution was built on four levels: data input, data analysis, data storage, and data output. The levels were interconnected using representational state transfer application programming interface (REST APIs), along with the annotation model, the ontology, and the database with annotated documents stored in the cloud. The initial ontology for an IoT cybersecurity field was developed using Protégé 5 and WebProtégé. Furthermore, IBM Watson’s Knowledge Studio service was integrated with the aim of implementing a dictionary and creating a rule-based model starting from the developed ontology. The model created in Watson Knowledge Studio was used and applied to documents by connecting it to IBM Cloud Watson Discovery. The processes of training and validating the system are presented in Section 4. Section 5 describes a gateway that can scan an existing IoT network and can send the gathered data to the solution presented in Section 4. The gateway extracts data such as the technologies used, types of devices, versions, and vendors. The gateway automatically detects the connections of new IoT devices to the local network and saves the metadata into a local ontology. Every time it is necessary, the gateway sends the gathered data to the NER solution that then analyzes it, searches through the annotated documents, and returns relevant information about the specific components of the network. This information can be further used by security management experts to handle the IoT environment properly. The section also presents the advantages given by the current approach compared to other behavioral analysis systems that focus on the information exchanged in order to detect possible threats. Two uses cases are described—a house with a Samsung Smart TV connected to a WiFi router that is running Tizen OS and an Insteon Hub for controlling smart light bulbs and outlets. Section 6 presents the limitations of the current work, especially the ones caused by the usage of commercial software. Conclusions and future work are highlighted in the last section of this paper.

## 2. Related Works

Under the topic of cybersecurity for IoT systems, several authors conducted research on sub-topics such as security vulnerabilities in IoT systems and automatic detection of vulnerabilities along with cyber situation awareness conceptualization and implementation. The previous research has focused more on detecting security threats at the point of appearance as opposed to an investigation into certain IoT systems and components conducted using a priori analysis. For sustaining our original proposal, we present the main contributions to the current researched topic, highlighting the insufficiently explored, designed, implemented and tested approaches with consideration to cybersecurity IoT systems.

In Reference [8], a hybrid approach for intrusion detection was designed in the area of cloud computing. The authors used an ontology in order to detect intrusion, using an event correlation approach. As mentioned before, this type of implementation detects threats at the moment of appearance, in contrast to our proposed solution that uses the ontology for describing the current status of an IoT system for diagnostic purposes.

Other research conducted in works such as References [9,10,11], focused on using ontology-based security frameworks in cloud computing, IoT-based smart homes, or mobile devices for detection but not for prevention.

Closely related to our methodological approach, Reference [12] proposed a security risk management method in order to measure the security of the analyzed systems; however, this study limited the focus on risk management. The work uses an ontology-oriented knowledge base for information security.

To sustain the need of using, as an initial database, the list of IoT-oriented CVEs in our current proposal, numerous articles consider CVEs as main data source for vulnerabilities [5,13,14,15]. In Reference [14], the authors emphasize the need for a structured and trustworthy database of information regarding vulnerabilities, attacks, threats, countermeasures, and risks within the task of information security risk management processes. To achieve this, they introduced a taxonomy to classify and compare several data sources based on the type of information, integrability, timeliness, originality, type of source, and trustworthiness. The results of the study show that, in research and practice, specialists rely on a large variety of heterogeneous information security data sources, making it difficult to know, use, and integrate them in the process of risk management. Under the circumstances of this research, emphasizing our current approach, we propose an even deeper analysis and implementation of the prior mentioned sources of information security in the area of IoT systems.

In studies such as Reference [15] and Reference [16], several CVEs connected with IoT issues are addressed, without taking into consideration a methodological approach for gathering integrated information within this field. Our proposed framework integrates these aspects in an automatic manner, taking a further step toward creating an ontology of CVEs-based information.

In a study [17], the authors used the database of CVEs in order to compute a model for identifying different trends of new CVEs based on forecasts. Using an LDA (latent Dirichlet allocation), an unsupervised learning technique, the results obtained were given using a new classification system, called the topic model. This research adds valuable information to the current manual classification of CVEs, combining automated classification to possible prediction over time. Given the major contribution to the general study of vulnerabilities and exposures, the study uses all CVEs, without taking into consideration sub-topics of interest, such as IoT systems. Trend analysis of CVEs is also conducted in studies [18,19,20] that conclude that understanding the vulnerabilities trends is a key component of the risk management process. These works focus more on a statistical approach of trend evaluation, calculating the overall frequency and severity based on CVSS (common vulnerabilities scoring system) metrics. Our study concentrates on the CVEs that are relevant to IoT systems, using them as a training background for our proposed model.

Cyber situation awareness is a topic of great interest in the area of cybersecurity, as the main aspects are focused on being aware of the situation, on the impact of the attack and knowing how the situation can evolve. However, being aware of the quality and trustworthiness of the collected situation awareness information items and the knowledge-intelligence-decisions derived from these information items, as presented in Reference [21], is of great interest. This is also a major concern within our own current research paper. Gathering valuable information from the CVEs database offers trustworthiness and high quality of the input information in the area of the risk management process. Other studies, such as Reference [22] and Reference [23], discuss situation awareness in the area of cybersecurity, conducting research on the automated gathering of information from multiple heterogeneous information security data sources.

Another study focused on the topic of cybersecurity awareness, addressed the issue of implementing a framework for cybersecurity situation awareness based on data mining as guidance and technical support for the entire situation awareness procedure [24]. The limitations of the proposed framework are given by the lack of testing in a real environment and by the evaluation of the integrated models. The central point of the research uses a data source of knowledge mining collected from experimental cyberspace or known data sets. A correlation analyzing component processes cybersecurity events in real-time based on known patterns. This research addressed as well the issue of real-time security events analysis in contrast to our proposed work that extracts, in an IoT-oriented domain, an automatic selection of the information found within CVEs relevant to each particular situation. A more closely related work to our proposal is the one conducted in Reference [25], in which an ontology that modeled vulnerabilities within IoT systems was created. The core solution uses Shodan and Censys search engines with the aim of reducing the number of aggregated results during the search and increasing the relevance of the obtained results. Shodan searching engine interrogates device ports and grabs banners, resulting in public internet protocol (IP) addresses. Censys search engine collects data by scanning IP addresses.

Articles in Reference [7] and Reference [26] describe a cognitive cybersecurity assistant which uses a similar approach to the solution described in this section. However, they do not focus on IoT security in particular and do not take into consideration the relations between certain concepts. Other projects that use instruments similar to the ones described in this paper are available in References [27,28,29]. Reference [27] and Reference [28] propose models for obtaining the information in the medical field, extracting relevant data which can be used to make valuable inferences. Reference [29] proposes a model which extracts relevant information about shipping industry emails. Although the instruments and performance metrics are very similar to the ones used in this paper, there is a significant difference in our approach. All the papers mentioned have a relatively simple ontology from which they start the training process. The model proposed in Reference [28] contains five classes and the one described in Reference [29] only four classes, compared to the 17 classes modelled in our solution. Their main objective is to retrieve information only for a small subset of data in their domain. Instead, our approach is to identify as much relevant data as possible that is connected to IoT and IoT security.

Concluding our research on the current related work, the originality of the proposed solution is given by

- designing a framework for the automated extraction of information related to IoT found within the database of available CVEs using semantic analysis as an input stage for automatic context-based filtering within the CVEs database;
- extracting training data using a list of keywords in the form of a sub-set of CVEs associated with IoT systems;
- creating a domain-based ontology for vulnerability information within IoT systems;
- addressing optimization issues within the general concept of cyber situation awareness in order to integrate it into the process of security information risk management.

## 3. Security Vulnerabilities for IoT Technologies

The common vulnerabilities and exposures (CVE) system provides a reference method for publicly known vulnerabilities and exposures. The National Cyber-Security Federally Funded Research and Development Center (FFRDC), operated by MITRE Corporation, maintains the system, which is funded by the US Department of Homeland Security’s Cyber Security Division. The security content automation protocol uses CVEs, and CVEs are listed in the MITRE system as well as in the US National Vulnerability Database [6]. Within the CVE vulnerability database, all the important IT vulnerabilities reported so far are stored. Shortly after a new vulnerability is reported, it is documented and receives a new unique identifier in the CVE list. Currently, the CVE vulnerability base is a reference resource for cybersecurity specialists. CVE databases are available in Reference [6] and Reference [30]. At the time of writing this paper, there are 112,044 different CVEs, documented since 1999, and their number is steadily increasing [6].

Within this section, the most relevant IoT records were downloaded from the total number of CVEs available. Given the novelty of the IoT field, as well as the dynamism specific to modern IT technologies, CVEs were selected for the time frame 2012–2018. In order to select only relevant CVEs, keyword searches related to IoT standards, technologies, and products have been used. As it has been previously argued, these vulnerabilities are specific to the technologies dedicated to IoT and not specific to other technologies. Table 1 lists the main CVEs relevant to the IoT domain. These were grouped according to key terms specific to the IoT domain. There were also other key terms used in IoT, but they are not present in the table because no extra CVEs containing any of these terms were found [31]. The full version of this table is available in Appendix A (Table A1).

The obtained results contain 312 CVEs that are specific to the IoT domain, grouped by 33 key terms. Due to reasons related to space, the hyperlink of each identified CVE was not included. The full description of each CVE is available in Reference [6] and Reference [30]. The CVEs relevant to the IoT field were analyzed and used as data corpus for training the NER model presented in Section 5.

**Figure 1:**
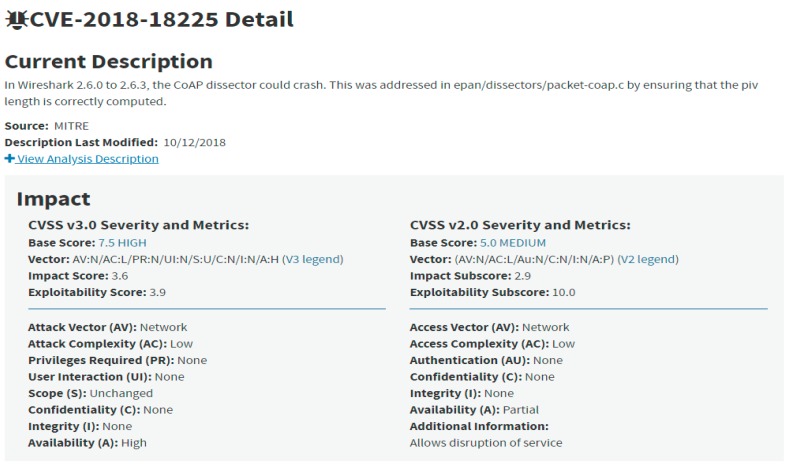
CVE-2018018225 details and common vulnerability scoring system (CVSS) scoring as displayed in Reference [6].

Figure 1 displays the details found within a CVE object, using the example of CVE-2018-18225. Each CVE contains a list of sections along with scores from the common vulnerability scoring system (CVSS), which is used for assessing and prioritizing each individual piece of vulnerability data. A short description in natural language of the CVE is presented, containing valuable information that is used in the next section of our proposed model for the automatic gathering of cybersecurity data from IoT systems.

## 4. Using NER for the Automatic Gathering of Cybersecurity Data Regarding IoT Devices

In this section, we propose an original solution designed to facilitate the gathering of information regarding the security of IoT systems. One of the authors of this paper (T.M.G.) implemented a similar solution which allows users to upload text documents, analyzes them, and automatically provides insights into cybersecurity issues, based on entity recognition instruments. The solution is called Cybersecurity Analyzer and is available in Reference [32]. The main contribution of the solution developed and described in this section is that it is specially designed for IoT, with an IoT-specific ontology, as well as an NER model designed for semantic text analysis of IoT security. The goal of the solution is to automatically monitor cybersecurity information relevant for IoT, filter and present it in an organized and structured framework based on user’s needs. The developed model is further connected with a gateway which provides data gathered from IoT devices, as described in Section 5. The solution returns the main possible vulnerabilities specific to the analyzed devices.

### 4.1. The Solution’s Architecture

**Figure 2:**
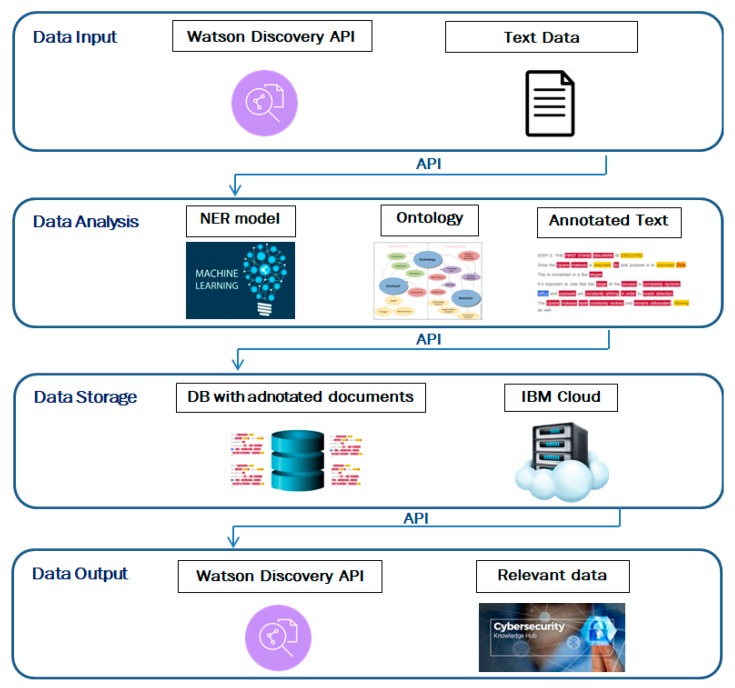
The solutions’ architecture with the four levels of data processing.

The software solution architecture is built on four levels, according to the functional requirements: (1) data input, (2) data analysis, (3) data storage, and (4) data output. Figure 2 illustrates the architecture of the solution.

The first level of the architecture handles data input. The solution can accept input text data, through a REST API (representational state transfer application programming interface). The documents entered into the model were obtained with the usage of a scraping component that automatically extracts text data from selected websites. The first two levels are connected through a REST API. Level 2 is designed for data analysis and comprehension. A domain ontology is used, over which an NER model specialized in semantic text analysis has been implemented. The model enriches the data with semantic annotations. The data storage level contains a database with relevant documents. These documents are annotated by the NER component from level 2. The last level offers the possibility to interrogate the documents database through a REST API. Advanced searches can be made based on keywords, entity types, or relation types. Each component of the architecture is described in detail below.

### 4.2. Choosing Technologies

The ontology was initially developed using Protégé 5 and WebProtege [33]. In order to implement the IoT security ontology, IBM Watson’s Knowledge Studio service was chosen [34]. It offers both facilities to implement a dictionary and create a rule-based model, as well as to develop a machine learning model with NER capabilities, starting from the developed ontology. Due to commercial reasons, no details about how the custom machine learning algorithm is implemented behind the scenes are publicly available. However, we consider this a limitation that does not affect the overall added value of our proposed approach. Knowledge Studio is available in the cloud and can be accessed through two web interface modes or by connecting to the REST API. The following components were implemented: classes, relations between classes, entities, various lexical forms for the same entity, rules, and a series of predefined properties for the types of speech that every entity can have (such as the degree of generality).

The models implemented in Watson Knowledge Studio can be used and applied to documents by connecting them with another service available in the IBM Cloud called Watson Discovery [35]. Watson Discovery allows the use of the developed NER model by integrating it with other services. During the processes of developing and validating the entity recognition model in Knowledge Studio and Knowledge Discovery, we used both interfaces and REST APIs. The first was used due to the ease of working directly in the interface. Working with REST APIs was useful when automation was required. For connection to REST APIs, Postman [36] has been used, as well as custom made scripts.

### 4.3. The Ontology

**Figure 3:**
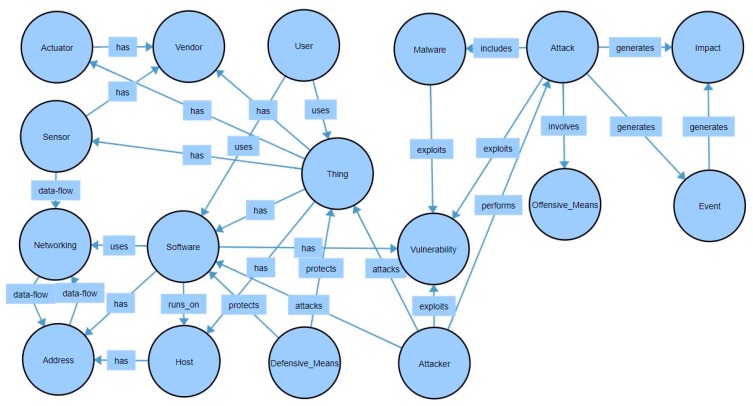
The structure of the ontology used for NER (classes and relations).

Several articles that describe ontologies for IoT security, such as References [7,10,18,37], use various approaches. The ontology described in this section is based on the ones developed in References [7,18,37] and was designed to facilitate the further development of the entity recognition model. Figure 3 illustrates the classes of the developed ontology as well as the relations between them.

The Ontology contains 17 classes as follows: Actuator, Address, Attack, Attacker, Defensive Means, Event, Host, Impact, Malware, Networking, Offensive Means, Sensor, Software, Thing, User, Vendor, and Vulnerability. Both the classes and the relations between them can be observed in the figure above.

### 4.4. Developing the NER Model

#### 4.4.1. From Ontology to Entity Recognition

The term “named entity recognition” refers to a subtask of natural language processing which aims to recognize (without being limited to) information units such as names, including person, organization, and location names and numeric expressions including time, date, money, and percentage expressions [38]. There are multiple ways of performing NER, such as supervised learning, semi-supervised learning, and unsupervised learning [38]. Watson Knowledge Studio arguably uses a semi-supervised learning approach by combining manually annotated data with context-based knowledge extraction.

The idea of implementing an NER model originated amid constraints on ontology-based solutions. Starting from the elements defined by the ontology and using specific machine learning algorithms, a well-trained model offers two important advantages:

- It can identify new forms of the instances that are already defined in the ontology;
- It can identify new class instances similar to the existing ones.

In this regard, the Watson Knowledge Studio tool was used. The classes of the ontology became the classes of the Knowledge Studio dictionary and each class had assigned a series of entities. Each entity had various surface forms. The relations between classes were defined, and these can be observed in Figure 3.

#### 4.4.2. Training the Model

The main data training source consisted of the CVEs described in Section 3 (70% of the corpora). The rest of the data corpus were research articles (10%), technical reports (10%), as well as various online articles that we considered to be relevant (10%). The training set contained 19,000 tagged entities and over 43,000 tokens and was split according to the Knowledge Studio default percentages (70% train data, 23% test data, and 7% blind data).

**Figure 4:**
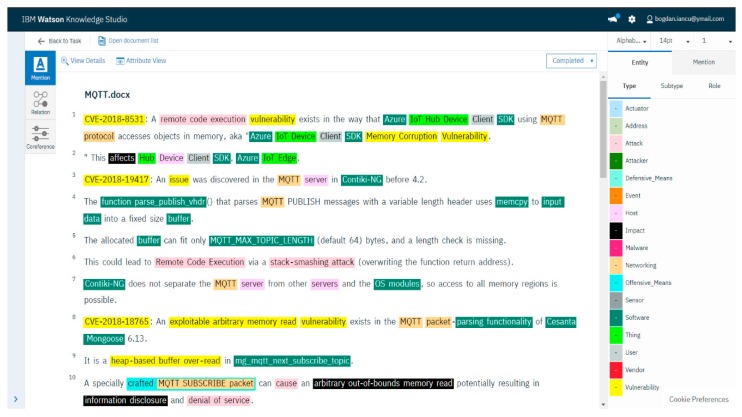
A screen capture of the training process for entity recognition from Knowledge Studio.

The training process was performed using the Knowledge Studio interface. The use of the ontology model facilitated the training process. For documents loaded to be trained, the ontology-based model was first applied, identifying a large number of relevant instances. Documents were first automatically annotated using dictionaries and then manually annotated for parts that were not covered. Classes, entities, and the relations between them were identified. Figure 4 illustrates a screen capture taken during the training process.

**Figure 5:**
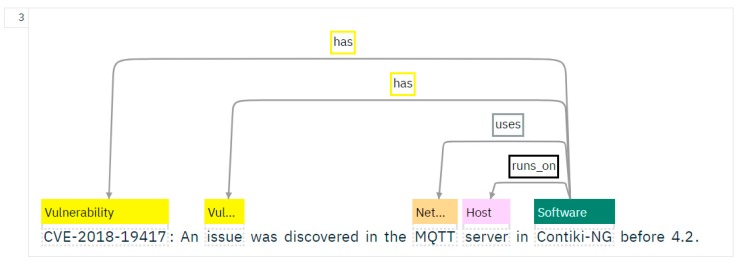
Training process from Knowledge Studio for relations recognition.

Figure 5 illustrates the relations for one paragraph of the training data.

#### 4.4.3. Data Storage

The model implemented in Watson Knowledge Studio is used by connecting it to another service available in the IBM Cloud called Watson Discovery. Watson Discovery allows the use of the developed NER model via integration with other services. For the proposed prototype, Watson Discovery can be used in two ways:

1. As an artificial intelligence solution that connects with the Knowledge Studio service and performs entity recognition based on models created in Knowledge Studio. Within Watson Discovery, document feeds can be automatically uploaded via a REST API. These are enriched with annotations and, for each document, a JSON with metadata is returned.
2. Both as a cognitive text analysis solution (according to point 1) and as a storage solution for uploaded documents. Documents can be stored directly in Watson Discovery to be used at an aggregate level directly in the IBM Cloud platform. This makes work easier, as there is no need to develop new storage components. On the other hand, the IBM Cloud service is commercial, and the storage of a large volume of documents involves some costs. For this article, no costs were involved, as we used free accounts created by IBM for research purposes.

After the model was trained, documents containing the 312 identified CVEs were introduced in order to be enriched by the model, as well as other documents regarding IoT security. These documents are stored on IBM Watson Discovery cloud platform and can be accessed via a REST API. Section 5 discusses certain application scenarios in which queries are performed in order to filter, select, and return only relevant documents.

#### 4.4.4. Validating the Model

In order to validate the results, the main performance indicators specific to the NER domain proposed by CoNLL 2018 [39] were taken into account: F1 score, Precision, and Recall. The indicators were calculated for the main two functionalities of the solution: entity extraction and relation extraction. The main formulas are:

(1) $$\mathrm{Precision}=\frac{\mathrm{True}\;\mathrm{Positive}}{\mathrm{True}\;\mathrm{Positive}+\mathrm{False}\;\mathrm{Positive}},$$

(2) $$\mathrm{Recall}=\frac{\mathrm{True}\;\mathrm{Positive}}{\mathrm{True}\;\mathrm{Positive}+\mathrm{False}\;\mathrm{Negative}}$$

(3) $$F1\;\mathrm{score}=\frac{2\times \mathrm{Recall}\times \mathrm{Precission}}{\mathrm{Recall}+\mathrm{Precission}}.$$

**Figure 6:**
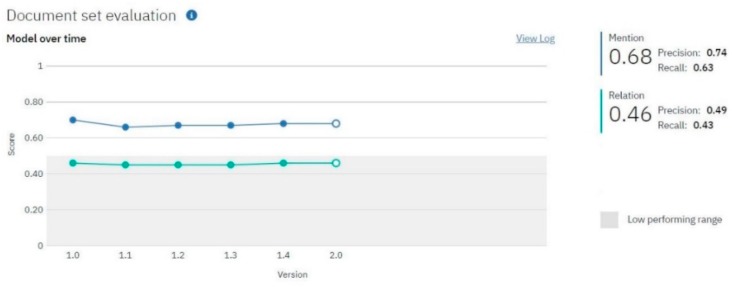
The evolution of the NER model and the obtained values for indicators according to Knowledge Studio.

Figure 6 illustrates the values of the F1 score for entity extraction and relation extraction, as well as the algorithm’s convergence for six consecutive iterations that were run on larger and larger datasets.

We consider that the high values of version 1.0 can be attributed to the fact that the first sets of training, test, and blind data were homogeneous, containing only CVEs relating to the IoT field. Once the training set was more diversified and training data contained different structures or approaches, the performances suffered a little decrease. The F1 score for entity recognition is 0.68, which validates the NER model. The precision is 0.74 and the recall 0.63. Considering the high number of classes in our model, we consider that a 0.74 precision value indicates that the model is fairly accurate. A lower recall value usually indicates that the model can be improved by increasing the amount of training data. A future training session will be performed to increase the F1 score of the relation recognition, which at this point is below 0.5, as can be observed in Figure 6. It is important to mention that, in the current configuration, the gateway described in Section 5 uses only entity recognition functionalities; therefore, the low F1 score for relation extraction is not particularly relevant to the current project.

Table 2 illustrates a comparison in terms of performances obtained by other projects which used similar technologies. The project in Reference [27] has a very low F1 score, as the authors stated that the work is still in the incipient phase. The projects in Reference [28] and Reference [29] obtained similar F1 scores for entity recognition as our work. However, it is important to point out that our model has a larger number of classes and relations, which significantly increases its complexity. As mentioned below, our approach was relatively different to the ones from other projects since we designed the entity recognition model to be able to annotate any relevant aspects connected to IoT and IoT security, which is a fairly large and diversified set of data. Superior results to those obtained with our approach were reported in Reference [7], which used the Stanford NER algorithm to identify cybersecurity-related concepts from heterogeneous data sources. The main reason for the superior result is most likely due to the lower number of classes and the larger domain (cybersecurity in general vs cybersecurity in IoT in our approach) used in Reference [7]. This could be confirmed by similar results obtained by one of the authors of this paper, in Reference [32], upon tuning the proposed model to work with a larger data set in a general cybersecurity context.

In the future, we will continue to train the developed model in order to improve the F1 score for both entity recognition and relation extraction.

### 4.5. Data Output—API

The relevant documents are stored in the Watson Discovery database. Various searches can be made through the REST API. The solution returns only the documents connected with the specific characteristics of the IoT devices of interest. Thus, users can easily discover the most important security aspects of the IoT devices they are using. The results are returned in JSON format. The application example in Section 5 illustrates how the process works.

## 5. Semantic Security Gateway

The vulnerability data relevant to the IoT domain, obtained in the previous step, would be partially wasted if the final user of an IoT network was unable to access it in an easy and convenient way. To avoid this, this section proposes a semantic gateway model, that can be integrated into the IoT network and that can make use of the vulnerabilities indexed in the Watson Discovery database.

**Figure 7:**
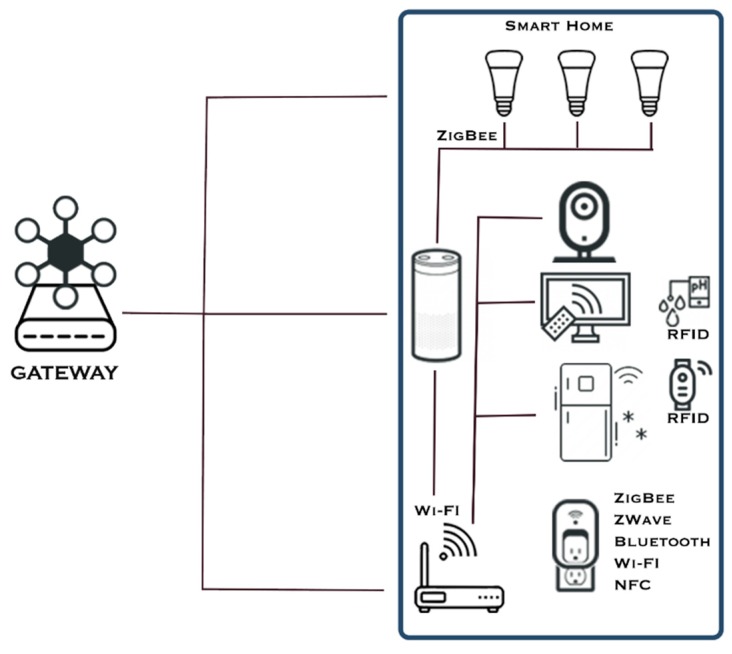
The data flow in the context of the gateway in a smart home environment (based on the architecture presented in Reference [31]).

A gateway device in an IoT network implements the single point of failure (SPOF) architecture by cutting the connection between the internet and the local IoT network in case of any incident [40]. In addition to this, it can analyze and filter the traffic between the internet and the local network and it can automatically manage the connection of new IoT devices to the local network and abstract the access to any of the sensors by automatically detecting the type of protocol needed for communication. However, the most important advantage of a gateway-based architecture is that, if one implements a security mechanism at this level, the entire local IoT network can be protected from exterior threats [31]. This is an important aspect because some of the IoT devices are too small or use a very simple communication protocol to facilitate the implementation of standard cybersecurity algorithms directly on their level [31]. Figure 7 presents the data flow from an IoT network in the context of a gateway installed in a smart home environment.

**Figure 8:**
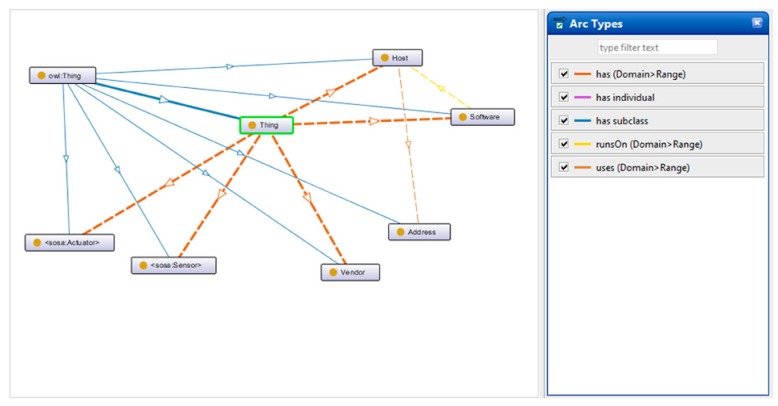
A simplified version of the ontology created on top of a Semantic Sensor Network (SSN) that models the local IoT network (screen capture from Protégé software).

The Semantic Sensor Network ontology (abbreviated SSN) is the W3C’s standard semantic implementation of the IoT domain. Based on the SOSA (sensor, observation, sample, actuator) ontology, it allows the modeling of entities such as sensors, actuators, observations, features of interest [3]. By using this ontology, one can model the local IoT network in a semantic format [41]. Even though the standard semantic technologies proposed by W3C are not yet supported by IBM Watson, this ontology will be interconnected with the entity recognition model obtained in the previous chapter via REST API calls. A screenshot of the taxonomy of the created ontology from the Protégé software is available in Figure 8.

The local ontology is saved only at the gateway level in an Apache Jena triplestore. As shown in [42], Apache Jena is one of the top triplestore solutions and it can be successfully installed on small devices that can play the role of the gateway, such as Raspberry Pi [43].

The advantage of storing local data in a semantic format is that this data can be linked to the Linked Open Data cloud in such a manner that the data exchanged in the local network can be understood not only by humans but also by machines. In order to avoid a security breach inside the gateway by exposing private data, only a fraction of the data will be publicly available, similarly to how solid pods work with one’s personal data [44]. In this manner, each and every user of the smart gateway can choose what type of information to share and with whom.

As presented earlier, the semantic gateway will act as a situation-aware system for the local IoT network. Even though there are solutions for behavioral analysis of the information exchange in order to detect possible threats, as presented in Reference [45], the disadvantage of this approach is that it works “a posteriori”. The attack needs to happen in order for the gateway to detect it. If no attack happens, the vulnerabilities can remain there waiting to be discovered. The strength of the approach proposed within the current research is that a semantic gateway, that is using context-aware searches in the CVEs database for possible security breaches, can identify them “a priori”. Based on the types of devices that are interconnected and on the metadata related to them (such as producer, software version, port number), the semantic gateway can inform the owner of the IoT network of possible exploits that are present in the network and suggest fixes for them.

Let us take the case of a simple house that has a Samsung Smart TV connected to a WiFi router that is running Tizen OS and also an Insteon Hub which is running firmware version 1012 to control some smart light bulbs and outlets.

When the user starts the scanning process, by using an application on his phone for example, the gateway will extract the needed information about the local infrastructure and will start to call the IBM Watson API to find possible security breaches based on the CVEs available and the meta-data related to the IoT devices connected to the network. Below two queries sent by the gateway through IBM Watson API are illustrated:

*(1)*  enriched_text.entities:(text:Samsung,type:Vendor),enriched_text.entities:(text:Tizen OS,type:Software);
*(2)*  enriched_text.entities:(text:Insteon Hub,type:Thing),enriched_text.entities:(text:”firmware version 1016”,type:Software),enriched_text.entities:(text:REST API).

**Figure 9:**
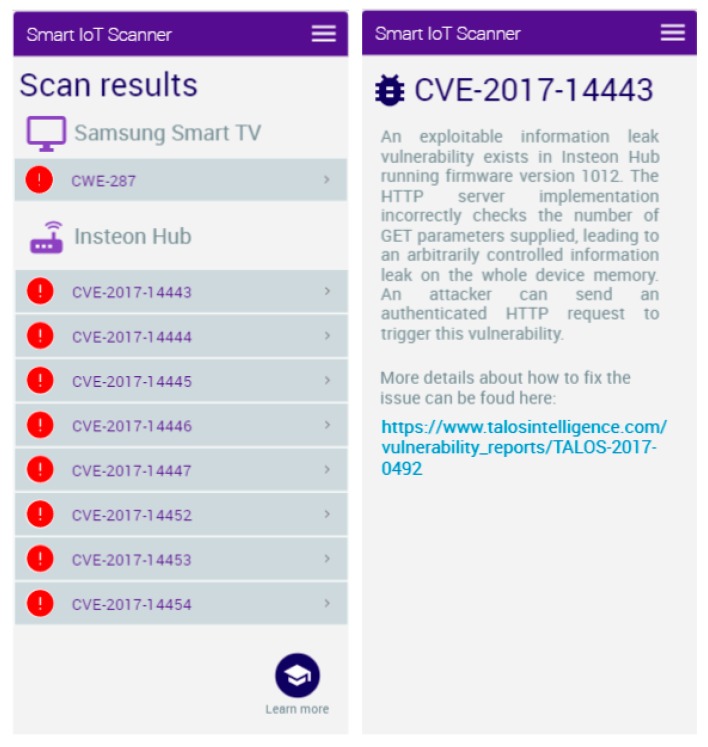
Example of results obtained by the smart scanning app in a smart home environment.

The first query searches for documents which include the entity “Samsung” in the context in which it belongs to the class “Vendor”, as well as the entity “Tizen OS” when it belongs to the class “Software”. Similarly, the second query searches for documents which discuss the “Insteon Hub“ when it belongs to the class “Thing “, and includes information about “firmware version 1016” of type “Software” and contains the entity “REST API”. Figure 9 presents the results obtained by the scanning app after performing the semantic queries illustrated above.

The solution searches through the enriched documents and returns the relevant data, which are sent to the mobile application. If any potential problems are found, the user is informed about the possible issues and what is causing them. Based on the metadata, the user can be prompted with solutions to the current problem, such as updating vulnerable software.

## 6. Limitations

This paper points out the robustness of the NER model and the reliability of the system. Although, we consider that our work has some limitations.

The main limitation is related to the use of a commercial tool to develop and implement the NER model. Using high-end natural language processing services, such as IBM Watson facilitates the development of performance cognitive analysis models. On the other hand, the commercial character of such products can limit the developer in terms of adjusting the solution. In addition, the source code of this type of product is a commercial secret, therefore certain analyses are not possible to conduct. However, the methodology used to calculate the performance indicators is an established one, used for many types of NER models [39]. IBM provides sufficient instruments and information in order to evaluate the model performances and confirm its validity.

Another limitation could be considered that the security of IoT systems can often be compromised due to traditional cybersecurity vulnerabilities, such as a general channel of data communication or a compromised PC connected to the system. The aim of this paper is to develop solutions able to recognize only IoT specific security flaws. In the future, our approach is to combine the model developed for IoT with general cybersecurity models, in order to provide more detailed information.

One final limitation is the timespan from the discovery of a new vulnerability to the moment when it becomes public in the CVEs database. This can be addressed in the future by including in the data corpus a list of cybersecurity-related websites that can be scrapped at some time interval.

## 7. Conclusions

The purpose of this paper—to enhance the process of diagnosing and detecting possible vulnerabilities within IoT systems—was achieved by combining an initial description of the cybersecurity in the IoT field in the form of a domain ontology (created using Protégé 5 and WebProtege), a selection of 312 CVEs oriented semantically in terms of the design description of cybersecurity in IoT, and an entity recognition system designed using IBM Watson Discovery. The output of the system can be interrogated using the framework proposed in Section 5, in which a semantic security gateway was implemented for the purpose of describing the components and communication protocols used within a specific IoT system. The gateway sends requests to the NER algorithm using a REST API with a structured query that describes the IoT system. Based on the performed query, the algorithm conducting the semantic analysis extracts and returns the highest prioritized CVEs within the database that correspond to main topics in the description of the IoT system.

Even though the implemented solution has some limitations presented in Section 6, it eases the process of documentation, selection, and staying up to date for the users of such systems e.g., security management experts. As a comparison to the other solutions presented in the related works section, our approach deals with the cybersecurity situational awareness topic within an IoT-domain-oriented framework. The originality of this paper is due to the use of a domain-oriented approach within IoT systems, achieving an entity recognition algorithm used for cybersecurity in IoT issues.

As a comparison to other solutions for behavioral analysis of the information exchanged, in order to detect possible vulnerabilities which have the disadvantage of an “a posteriori” approach, the strength of the proposed approach is that a semantic gateway that uses context-aware searches in the CVE database for possible security breaches works in an “a priori” manner. Once the system is scanned, it can output any problems encountered along with solutions to the current situation.

Future work will be focused on further testing the system with real data and IoT systems along with further training of the developed algorithm in order to improve its performance by comparing and analyzing the obtained results of the queries sent by the security semantic gateway. Along with this intensive testing and retraining of the entity recognition algorithm, other improvements will be made with regard to the data corpus size. A scraper for the automatic selection of relevant data over the internet facilitates the database dimension.

## Figures and Tables

**Table 1:** A subset of the common vulnerabilities and exposures (CVEs) relevant for the Internet of Things (IoT) domain.

No.	Key Term	Description	CVEs
1	Actuator	physical component	CVE-2014-9786, CVE-2014-9782, CVE-2014-9877
2	AMQP (Advanced Message Queuing Protocol)	protocol	CVE-2018-8119, CVE-2018-8030, CVE-2018-1298, CVE-2018-11087, CVE-2018-11050, CVE-2017-8045, CVE-2017-15702, CVE-2017-15701, CVE-2017-15699, CVE-2017-11408, CVE-2016-4974, CVE-2016-4432, CVE-2016-2173, CVE-2015-5240, CVE-2015-0862, CVE-2015-0203, CVE-2014-8711, CVE-2014-2814, CVE-2012-4458, CVE-2012-4446, CVE-2012-3467
3	Arduino	platform	CVE-2018-17614, CVE-2015-7833, CVE-2015-4590
4	Barcode	technology	CVE-2018-5221, CVE-2014-8672, CVE-2014-7897, CVE-2014-6869
5	Belkin WeMo	product	CVE-2018-6692, CVE-2013-6952, CVE-2013-6951, CVE-2013-6950, CVE-2013-6949, CVE-2013-6948
6	CoAP (Constrained Application Protocol)	protocol	CVE-2018-18225, CVE-2018-14367
7	Eclipse Kura	framework	CVE-2017-7649
8	Eclipse Mosquitto	framework	CVE-2018-12543, CVE-2017-9868, CVE-2017-9132, CVE-2017-9131, CVE-2017-7654, CVE-2017-7653, CVE-2017-7652, CVE-2017-7651, CVE-2017-7650
9	Embedded system	technology	CVE-2017-12823
10	HART (Highway Addressable Remote Transducer)	protocol	CVE-2018-16059, CVE-2015-6463, CVE-2015-3977, CVE-2014-9203, CVE-2014-9191, CVE-2013-2476

**Table 2:** Metric performances comparison between our work and other projects.

Project	F1 Score for Entity Recognition	Number of Classes	F1 Score for Relation Extraction	Number of Relations
[7]	0.8	7	Not Available	Not Available
[27]	0.49	5	0.19	Not Available
[28]	0.73	5	Not Available	Not Available
[29]	0.67	4	0.55	2
[32]	0.81	18	0.53	33
Our work	0.68	17	0.46	30

## Appendix A

**Table A1:** Common vulnerabilities and exposures (CVEs) relevant for the Internet of Things (IoT) domain grouped by key terms.

No.	Key Term	Description	CVEs
1	Actuator	physical component	CVE-2014-9786, CVE-2014-9782, CVE-2014-9877
2	AMQP	protocol	CVE-2018-8119, CVE-2018-8030, CVE-2018-1298, CVE-2018-11087, CVE-2018-11050, CVE-2017-8045, CVE-2017-15702, CVE-2017-15701, CVE-2017-15699, CVE-2017-11408, CVE-2016-4974, CVE-2016-4432, CVE-2016-2173, CVE-2015-5240, CVE-2015-0862, CVE-2015-0203, CVE-2014-8711, CVE-2014-2814, CVE-2012-4458, CVE-2012-4446, CVE-2012-3467
3	Arduino	platform	CVE-2018-17614, CVE-2015-7833, CVE-2015-4590
4	Barcode	technology	CVE-2018-5221, CVE-2014-8672, CVE-2014-7897, CVE-2014-6869
5	Belkin WeMo	product	CVE-2018-6692, CVE-2013-6952, CVE-2013-6951, CVE-2013-6950, CVE-2013-6949, CVE-2013-6948
6	CoAP	protocol	CVE-2018-18225, CVE-2018-14367
7	Eclipse Kura	framework	CVE-2017-7649
8	Eclipse Mosquitto	framework	CVE-2018-12543, CVE-2017-9868, CVE-2017-9132, CVE-2017-9131, CVE-2017-7654, CVE-2017-7653, CVE-2017-7652, CVE-2017-7651, CVE-2017-7650
9	Embedded system	technology	CVE-2017-12823
10	HART	protocol	CVE-2018-16059, CVE-2015-6463, CVE-2015-3977, CVE-2014-9203, CVE-2014-9191, CVE-2013-2476
11	Home automation	solution	CVE-2015-3610, CVE-2014-4892, CVE-2013-6952, CVE-2013-6951, CVE-2013-6950, CVE-2013-6949, CVE-2013-6948
12	Homekit	solution	CVE-2017-13903, CVE-2017-2434
13	IIoT	technology	CVE-2018-18396, CVE-2018-18395, CVE-2018-18394, CVE-2018-18394, CVE-2018-18393, CVE-2018-18392, CVE-2018-18391, CVE-2018-18390
14	Insteon	product	CVE-2018-3834, CVE-2018-3833, CVE-2018-3832, CVE-2018-12640, CVE-2018-11560, CVE-2017-5251, CVE-2017-5250, CVE-2017-16348, CVE-2017-16347, CVE-2017-16346, CVE-2017-16345, CVE-2017-16344, CVE-2017-16343, CVE-2017-16342, CVE-2017-16341, CVE-2017-16340, CVE-2017-16339, CVE-2017-16338, CVE-2017-16337, CVE-2017-16252, CVE-2017-14455, CVE-2017-14453, CVE-2017-14452, CVE-2017-14447, CVE-2017-14446, CVE-2017-14445, CVE-2017-14444, CVE-2017-14443
15	IoT	technology	CVE-2018-8531, CVE-2018-8479, CVE-2018-8119, CVE-2018-12163, CVE-2018-11682, CVE-2018-11681, CVE-2018-11629, CVE-2018-0270, CVE-2017-7911, CVE-2017-7243, CVE-2017-6780, CVE-2017-14913, CVE-2017-14912, CVE-2017-14911, CVE-2017-14910, CVE-2017-14906, CVE-2017-11010, CVE-2015-4080, CVE-2015-2247.
16	MQTT (Message Queuing Telemetry Transport)	protocol	CVE-2018-8531, CVE-2018-18765, CVE-2018-18764, CVE-2018-17614, CVE-2018-17614, CVE-2018-1684, CVE-2018-1684, CVE-2018-15323, CVE-2017-9868, CVE-2017-7651, CVE-2017-7650, CVE-2017-7296, CVE-2017-2895, CVE-2017-2894, CVE-2017-2893, CVE-2017-2892, CVE-2016-9877, CVE-2016-10523, CVE-2014-6116, CVE-2014-0923, CVE-2014-0922
17	NFC	technology	CVE-2018-7930, CVE-2017-2287, CVE-2017-2286, CVE-2017-2149, CVE-2017-17280, CVE-2017-17225, CVE-2017-15322, CVE-2017-0784, CVE-2017-0481, CVE-2016-3761, CVE-2015-8041, CVE-2015-4033
18	OSRAM Lightify lights	product	CVE-2016-5059, CVE-2016-5058, CVE-2016-5057, CVE-2016-5056, CVE-2016-5055, CVE-2016-5054, CVE-2016-5053, CVE-2016-5052, CVE-2016-5051
19	Philips Hue	product	CVE-2017-14797
20	QR code	technology	CVE-2018-3900, CVE-2018-3899, CVE-2018-3898, CVE-2017-7696, CVE-2014-8672, CVE-2014-3651, CVE-2014-0239, CVE-2013-4872
21	RFID	technology	CVE-2018-5304, CVE-2018-5303, CVE-2018-4833, CVE-2015-6839, CVE-2013-0656
22	RPL	protocol	CVE-2014-3405
23	Sensor	physical component	CVE-2018-9276, CVE-2018-19204, CVE-2018-14891, CVE-2018-14890, CVE-2018-14889, CVE-2018-11399, CVE-2018-0453, CVE-2017-6798, CVE-2017-15814, CVE-2017-15008, CVE-2017-0709, CVE-2017-0582, CVE-2017-0527, CVE-2017-0526, CVE-2017-0519, CVE-2017-0518, CVE-2017-0517, CVE-2016-9568, CVE-2016-4038, CVE-2016-3934, CVE-2016-3903, CVE-2016-3798, CVE-2016-2398, CVE-2016-2311, CVE-2016-0866, CVE-2016-0865, CVE-2016-0864, CVE-2016-0863, CVE-2015-7743, CVE-2015-2878, CVE-2015-0739, CVE-2014-9890, CVE-2014-9877, CVE-2014-9868, CVE-2014-9866, CVE-2014-9786, CVE-2014-9783, CVE-2014-9782, CVE-2014-2362, CVE-2014-2361, CVE-2014-2360, CVE-2014-2359, CVE-2013-6124, CVE-2013-5321, CVE-2013-1219, CVE-2012-4621
24	Sensors	physical components	CVE-2018-5401, CVE-2018-1000044, CVE-2018-0453, CVE-2017-18303, CVE-2017-12879, CVE-2016-2813, CVE-2014-2379, CVE-2014-2378, CVE-2013-1243, CVE-2012-3901, CVE-2012-3899
25	Smart home	solution	CVE-2018-9162, CVE-2018-15125, CVE-2018-15124, CVE-2018-15123, CVE-2017-5249, CVE-2014-4892
26	Smartgrid	solutions	CVE-2016-0866, CVE-2016-0865, CVE-2016-0864, CVE-2016-0863
27	Smarthome	solution	CVE-2017-2704
28	SmartThings	product, hub	CVE-2018-3927, CVE-2018-3926, CVE-2018-3925, CVE-2018-3919, CVE-2018-3918, CVE-2018-3917, CVE-2018-3916, CVE-2018-3915, CVE-2018-3914, CVE-2018-3913, CVE-2018-3912, CVE-2018-3911, CVE-2018-3909, CVE-2018-3908, CVE-2018-3907, CVE-2018-3906, CVE-2018-3905, CVE-2018-3904, CVE-2018-3903, CVE-2018-3902, CVE-2018-3897, CVE-2018-3896, CVE-2018-3895, CVE-2018-3894, CVE-2018-3893, CVE-2018-3880, CVE-2018-3879, CVE-2018-3878, CVE-2018-3877, CVE-2018-3876, CVE-2018-3875, CVE-2018-3874, CVE-2018-3873, CVE-2018-3872, CVE-2018-3867, CVE-2018-3866, CVE-2018-3865, CVE-2018-3864, CVE-2018-3863, CVE-2018-3856
29	Vehicle	physical component	CVE-2018-18071, CVE-2017-1000474, CVE-2016-9337, CVE-2016-2354, CVE-2015-5611
30	Vehicles	physical component	CVE-2018-9322, CVE-2018-9320, CVE-2018-9318, CVE-2018-9314, CVE-2018-9313, CVE-2018-9312, CVE-2018-9311, CVE-2018-18070, CVE-2018-16806, CVE-2017-9647, CVE-2017-9633, CVE-2017-14937
31	Wearable	technology	CVE-2017-17773
32	Wink	product	CVE-2017-5249
33	Zigbee	protocol	CVE-2018-3926, CVE-2016-5058, CVE-2016-5054, CVE-2016-2398, CVE-2016-1562, CVE-2015-8732, CVE-2015-6244
